# Three new species of *Pilea* (Urticaceae) from limestone karst in China

**DOI:** 10.3897/phytokeys.19.3968

**Published:** 2012-12-28

**Authors:** Alex K. Monro, Y. G. Wei, C. J. Chen

**Affiliations:** 1 Department of Life Sciences, The Natural History Museum, London, SW7 5BD, United Kingdom; 2 Guangxi Institute of Botany, Chinese Academy of Sciences, Guilin, Guangxi 541006, People’s Republic of China; 3 Chinese National Herbarium, Institute of Botany, Chinese Academy of Sciences, Beijing 100093, People’s Republic of China; 4 The Herbarium, The Royal Botanic Gardens, Kew, Richmond, Surrey, TW9 3AB, United Kingdom

**Keywords:** Urticaceae, Pilea, China, Guizhou, Yunnan, limestone karst, caves

## Abstract

Three hitherto undescribed species of *Pilea* (Urticaceae) from limestone karst in China are described and illustrated. Affinities of the species are discussed and Global Species Conservation Assessments presented. The new species are *Pilea cavernicola* A.K. Monro, C.J. Chen & Y.G. Wei, **sp. nov.** (Vulnerable) which most closely resembles *Pilea scripta* (Buch.-Ham. ex D.Don) Wedd. and *Pilea gracilis* Handel-Mazzetti, *Pilea shizongensis* A.K. Monro, C.J. Chen & Y.G. Wei, **sp. nov.** (Endangered) which is most similar to *Pilea aquarum* Dunn and *Pilea guizhouensis* A.K. Monro, C.J. Chen & Y.G. Wei, **sp. nov.** (Vulnerable) which resembles *Pilea boniana* Gagnep. and *Pilea rubriflora* C. Wright mostclosely.

## Introduction

*Pilea* Lindley (1821: tab. 4) is the largest genus in the Urticaceae comprising ca 715 species (Monro, 2004) worldwide distributed throughout the tropics, subtropics and temperate regions (with the exception of Australia, New Zealand and Europe). Southeast Asia is the centre of morphological and phylogenetic diversity for *Pilea* whilst the Greater Antilles and Andean countries are the centres of species diversity (Monro, 2006). *Pilea* is easily distinguished from other Urticaceae by the combination of opposite leaves and a single, ligulate, intrapetiolar stipule in each leaf axil and pistillate flowers with a 3-5 parted asymmetrical perigonium. The majority of species are succulent herbs, epiphytes or small shrubs growing in heavy shade at altitudes between 1000 and 3000 m above sea-level. Within China 81 species are recognised (Chen and Monro 2003, Chen and Monro 2007) and of these 32 are endemic.

As part of ongoing research into the diversity of cave-dwelling Urticaceaethe authors undertook four field trips to SW China in 2009 and 2010. Collecting wasfocused on the karst area in Guangxi, Guizhou and Yunnan and caves, stoneforests (karst cockpit formations), gorges and natural forest were sampled. During the course of these collecting trips seven collections (not all in caves) corresponded to unknown species of *Pilea*, three of which are described here. Their affinities are discussed and position within Weddell’s (1869) and Chen’s (1982) subdivision of the genus indicated, which although not phylogenetic, is based on the most comprehensive world-wide treatment of the genus.

Collecting in Karst is difficult as areas away from roads are sparsely populated and the terrain is steeply dissected and difficult to travel across. A consequence of this is that there are relatively few collections from such areas and undescribed species are frequently represented by very little material. Describing species based on a single or only two collections is problematic as there is no estimate of variation within the species and so the risk of over recognising species is greater. This is compounded in Floras such as China’s where there has been a tradition of describing species from single collections resulting in a situation whereby the most closely related species may also be known from a single collection. Despite the above we have decided to describe one of the species in this manuscript based on a single collection as we feel that, given China’s fast changing landscape and the fragility of many of the localities, to describe the species now affords the best hope for their conservation.

## Methods

Herbarium specimens were compared with collections at IBK, BM and K and with scanned images of the collections at PE (http://pe.ibcas.ac.cn/herbinfor/vhtypequery.aspx ) and HK (http://www.hkherbarium.net/Herbarium/tcc_pop.aspx ) using the Flora of China. A morphological species concept developed during the course of previous taxonomic research on Pilea (Monro 2001 and 2006) was employed to delimit and compare taxa. Material was examined under a Wild M3C binocular microscope and Planapo lens at X64 to X400 magnifications.

In the case of cave habitats, photosynthetically active radiation (PAR) was recorded. Observations of PAR were made at several points in the caves associated with living plantsand expressed as micro moles per m2 per second (mmol/m2/sec) using a Skye instruments 180° panorama light meter for each point. Where available these observations are included in the Distribution section of the species descriptions.

Conservation assessments were undertaken using IUCN (2001) criteria B & D. Species distributions were plotted on Google Earth and the nature of the vegetation cover, urbanisation and road proximity surrounding sites, combined with observations in the field, were used as indicators of plausible future threats.

## Taxonomic treatment

### Pilea cavernicola

A.K. Monro, C.J. Chen & Y.G. Wei sp. nov.

urn:lsid:ipni.org:names:77123888-1

http://species-id.net/wiki/Pilea_cavernicola

**Figure 1.**
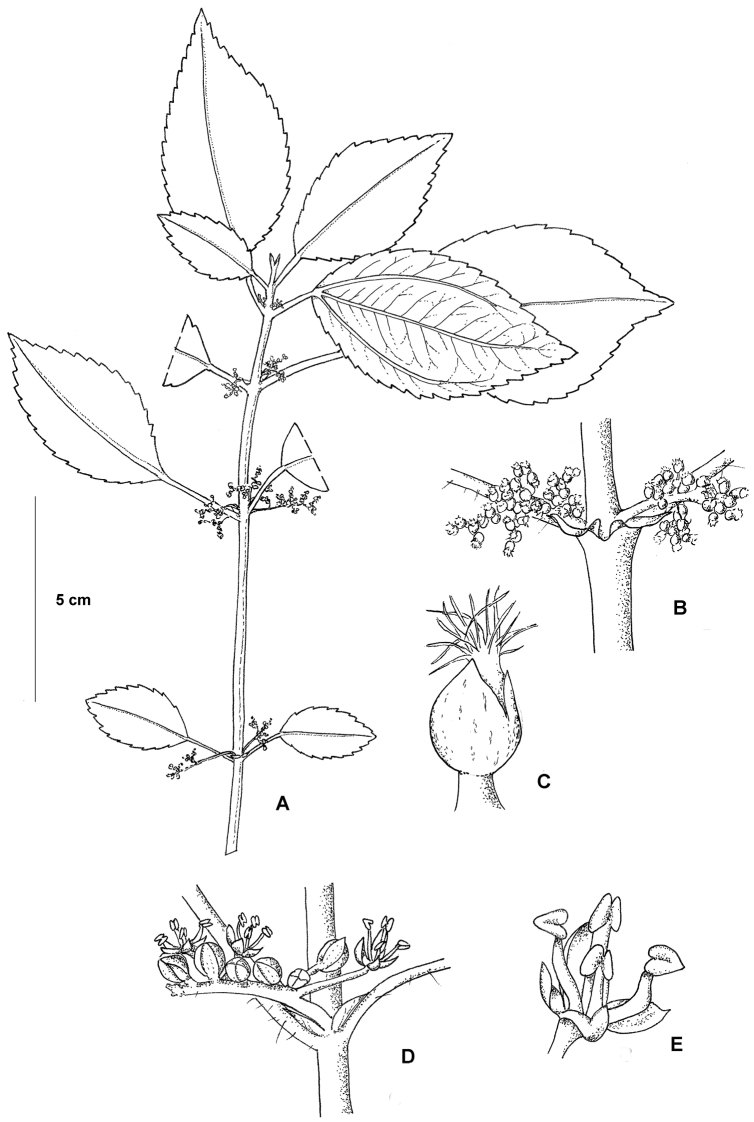
*Pilea cavernicola*. **A** Habit **B** Close up of a pistillate inflorescence **C** Close up of a pistillate flower **D** Close up of staminate inflorescence **E** Close up of staminate flower. Based on Monro & Wei 6669.

**Figure 2.**
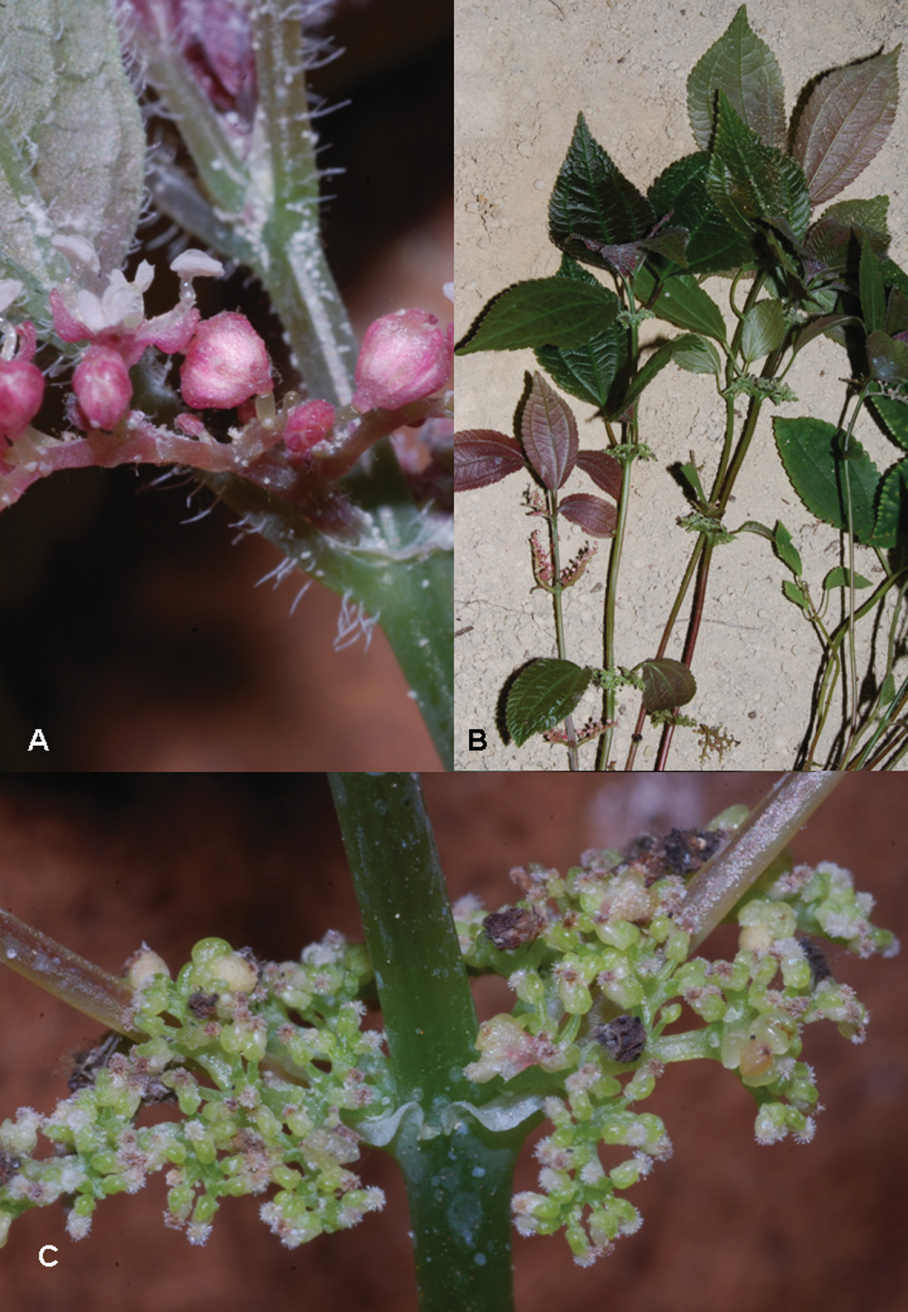
*Pilea cavernicola*. **A** Staminate flowers B Habit showing cave floor substrate in the background C pistillate inflorescence and flowers. Images of *Monro & Wei 6669*.

**Figure 3.**
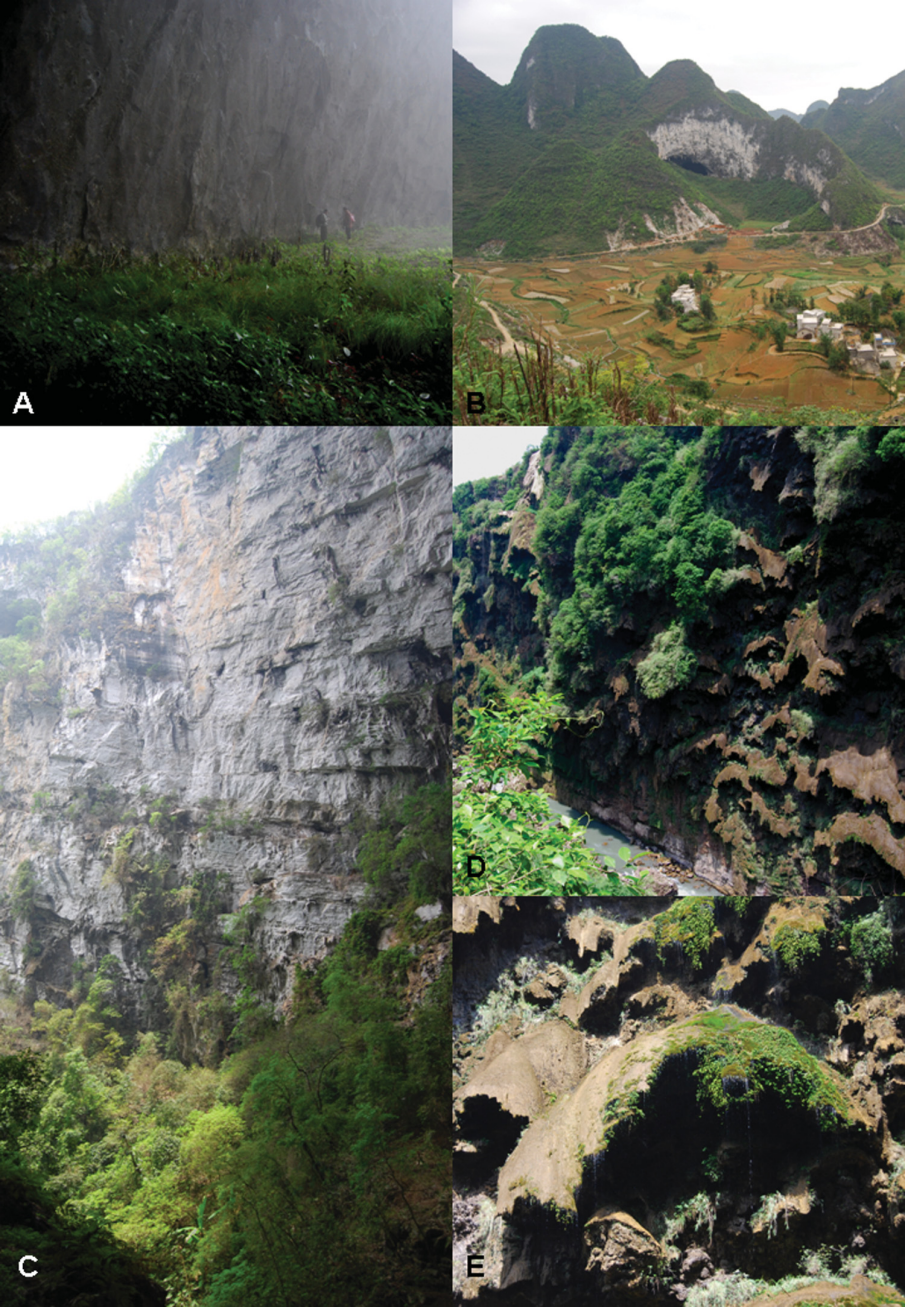
Habitats ofspecies described in this manuscript. **A, B** *Pilea cavernicola*, Yangzi cave, Fengshan County.Locality for *Monro & Wei 6669* **A** Interior of cave **B** Exterior view of the cave **C** Habitat of*Pilea shizongensis*, Feng Huang Gu gorge, Shizong County. Locality for *Monro & Wei 6727* **D, E** Habitat of *Pilea guizhouensis*, petaloid travertine wall of Malinghe Gorge **E** close-up of petaloid travertine formation, Locality for *Monro & Wei 6715*.

Figs 1 A–E , 2 A–C , 3 A–B

#### Diagnosis.

Most similar to *Pilea scripta* from which it can be distinguished by the shorter stems, ovate rather than elliptic or oblong leaves, stipules with auriculate bases rather than deltate ones, the staminate tepals not ribbed and the sub-compressed elliptic rather than ovoid achenes with smooth non verrucose surfaces.

#### Type.

**China.** Guangxi: Fengshan County, Paoli Town, Yangzi cave, 490 m, 024°23'22.2"N, 107°03'59.1"E (DMS), 9 May 2010, *A. K. Monro & Y.G. Wei 6669* (holotype: IBK; isotypes: BM001001214, MO, PE).

#### Description.

Herb to 50 cm, terrestrial. Stems erect, drying brown, maroon to green when fresh, glabrous or pubescent at the nodes and towards the base, where pubescent the hairs 1.0 mm, erect, crooked, cystoliths fusiform, the internodes 23–300 × 1.5–2.5 mm, angulate to square in cross-section, striate. Stipules 2.5–4.0 mm, auriculate-ovate, drying brown. Leaves petiolate, distichous; petioles at each node subequal or unequal by ratio 1:1.1–2.8, 12–33 mm, pubescent or glabrous, where pubescent the hairs 1.0-1.25 mm, erect, weakly curved or crooked; laminae at each node equal or subequal, 26–90 × 12–46 mm, ovate, subchartaceous; 3-nerved, the lateral nerves visible for 2/3 or more of the lamina length, secondary nerves 7–11 pairs, borne 60–75° to the midrib, weakly curved; upper surface drying dark brown, green or bronze when fresh, glabrous, cystoliths densely scattered, less than 0.125 mm, elliptic and punctiform, midrib and secondary nerves sunken; lower surface drying brown to dark brown, pale green or flushed bronze-purplish when fresh, pubescent, the hairs 0.75–1.0 mm, appressed, weakly curved, eglandular; base symmetrical, cuneate or weakly decurrent; margin serrate, the basal 1/8–1/4 entire; apex symmetrical, cuspidate. Inflorescences 4–8 per stem, unisexual, staminate and pistillate inflorescences synchronous, born on separate stems; bracts 0.75 mm; bracteoles 0.5 mm. Staminate inflorescences 2 per axil, 17–22 mm, bearing 45–90 flowers in a loose cyme; peduncle 1/4 or less inflorescence length, 0.75 mm in diameter, glabrous, occasionally with cystoliths present; pedicels 0.50–1.5 mm, glabrous. Staminate flowers 1.5 × 1.5 mm immediately prior to anthesis, green-brown; tepals 4, 1.75 mm, valvate, fused for their basal 1/3, elliptic, glabrous, the subapical appendage less than 0.25 mm, corniculate, glabrous; stamens 4. Pistillate inflorescences 1 or 2 per axil, 8–13 mm, bearing 150–300 flowers in a loose cyme; peduncle 1/4 to 1/3 inflorescence length, 0.75 mm in diameter, densely covered in cystoliths, cystoliths punctiform, glabrous; pedicels 0.25–0.75 mm, glabrous. Pistillate flowers 0.50–0.75 mm, tepals 3, unequal, glabrous, adaxial tepal 0.50–0.75 mm, oblong or ovate, the dorsal tepal appendage 0.50–0.75 mm, oblong, markedly thickened almost hood-like; the lateral tepals 0.375–0.50 mm, asymmetrically ovate. Infructescences 8–13 mm; peduncle 1/4 to 1/3 infructescence length; achenes 0.75× 0.675 mm, sub compressed, asymmetrically ellipsoid, the abaxial margin very narrowly thickened.

#### Distribution.

North West Guangxi Province, ca 500–1000 m, caves in limestone karst, growing at any point from the back to the entrance of the cave, PAR 0.02-1.39 mmol/m2/sec (ca 0.04-2.78 % full daylight).

#### Etymology.

The species name refers to the cave-dwelling habit of this species.

#### Paratypes.

CHINA: Guangxi Province: Fengshan County, Paoli Town, Sidui Village, Xibi cave, 740 m, 024°24'38.5"N, 107°04'53.5"E (DMS), 8 May 2010, *A. K. Monro & Y.G. Wei 6654* (IBK, BM001001215, PE, MO).

#### Discussion.

Comparison of the holotype and paratype material with type specimens from the herbaria listed in the methods section recovered *Pilea scripta* (Buch.-Ham. ex D.Don) Wedd. and *Pilea gracilis* Handel-Mazzetti as most similar to *Pilea cavernicola*. *Pilea scripta* can be distinguished from *Pilea cavernicola* based on stem height, leaf shape, stipule shape, staminate tepal morphology and achene morphology as summarised in Table 1. *Pilea gracilis* can be distinguished from *Pilea cavernicola* based on leaf shape, stipule morphology, staminate and pistillate inflorescence morphology and achene morphology as summarised in Table 2.

Pilea cavernicola falls within Weddell’s (1869) Dentatae-Gerontogeae subdivision and Chen’s (1982) Urticella Section of the genus.

**Conservation status.** Using IUCN criteria (IUCN 2001) *Pilea cavernicola* is considered Vulnerable (VU). *Pilea cavernicola* is known from only two localities (IUCN criteria D2, number of locations <5). At these localities the populations of this species comprises ca 100-200 mature individuals (IUCN criteria D1, number of mature individuals <1000). Using the IUCN methodology our Global Conservation Assessment for *Pilea cavernicola* is Vulnerable (VU)based on criteria D1 and D2: population size and number of locations combined with a plausible future threat that could drive this taxon to Endangered in a very short time. Plausible threats include the location of both caves at the edge of agricultural land, the use of the entrance of one of the cave localities (Yangzi cave) to cultivate medicinal plants (*Corydalis* sp.), requiring the terracing and tilling of the substrate. In addition mining is growing rapidly in the whole region and any localities close to roads are vulnerable to exploitation.

**Table 1.** XX

**Characters**	*Pilea cavernicola*	*Pilea scripta*
Stem height	to 50 cm	to 1.5 m
Leaf shape	ovate	elliptic or oblong-lanceolate
Stipule morphology	ovate with conspicuous auriculate base	triangular with deltate base
Staminate tepal morphology	not conspicuously ribbed	conspicuously ribbed
Achene morphology	sub compressed asymmetrical ellipsoid, the surface smooth	compressed asymmetrical ovoid, the surface verrucose

**Table 2.** XX

**Characters**	*Pilea cavernicola*	*Pilea gracilis*
Leaf shape	ovate, apex cuspidate	elliptic or elliptic-lanceolate, apex acuminate or caudate-acuminate
Stipule morphology	ovate with conspicuous auriculate base, 2.5–4.0 mm	triangular with deltate base, ca 1 mm
Staminate inflorescence	17–22 mm	20–50 mm
Pistillate inflorescence	8–13 mm	20–50 mm
Achene morphology	the surface smooth	the surface verrucose or verrucose-spinulose

### Pilea shizongensis

A.K. Monro, C.J. Chen & Y.G. Wei sp. nov.

urn:lsid:ipni.org:names:77123889-1

http://species-id.net/wiki/Pilea_shizongensis

**Figure 4.**
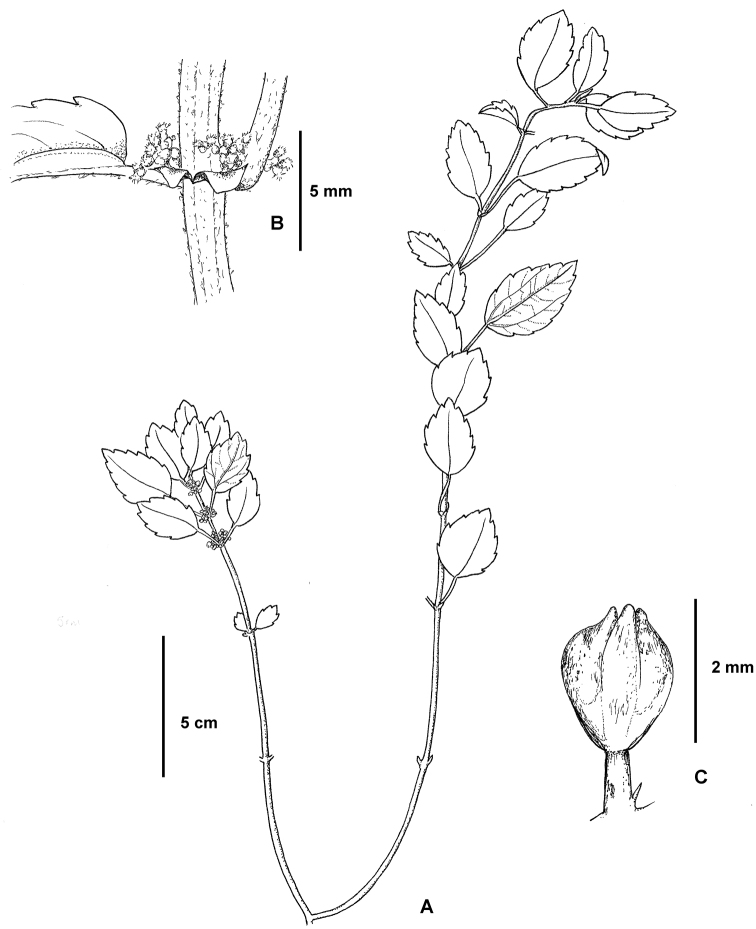
*Pilea shizongensis*. **A** Habit **B** Close up of pistillate inflorescence **C** Close up of a staminate flower. Based on *Monro & Wei 6727*.

**Figure 5.**
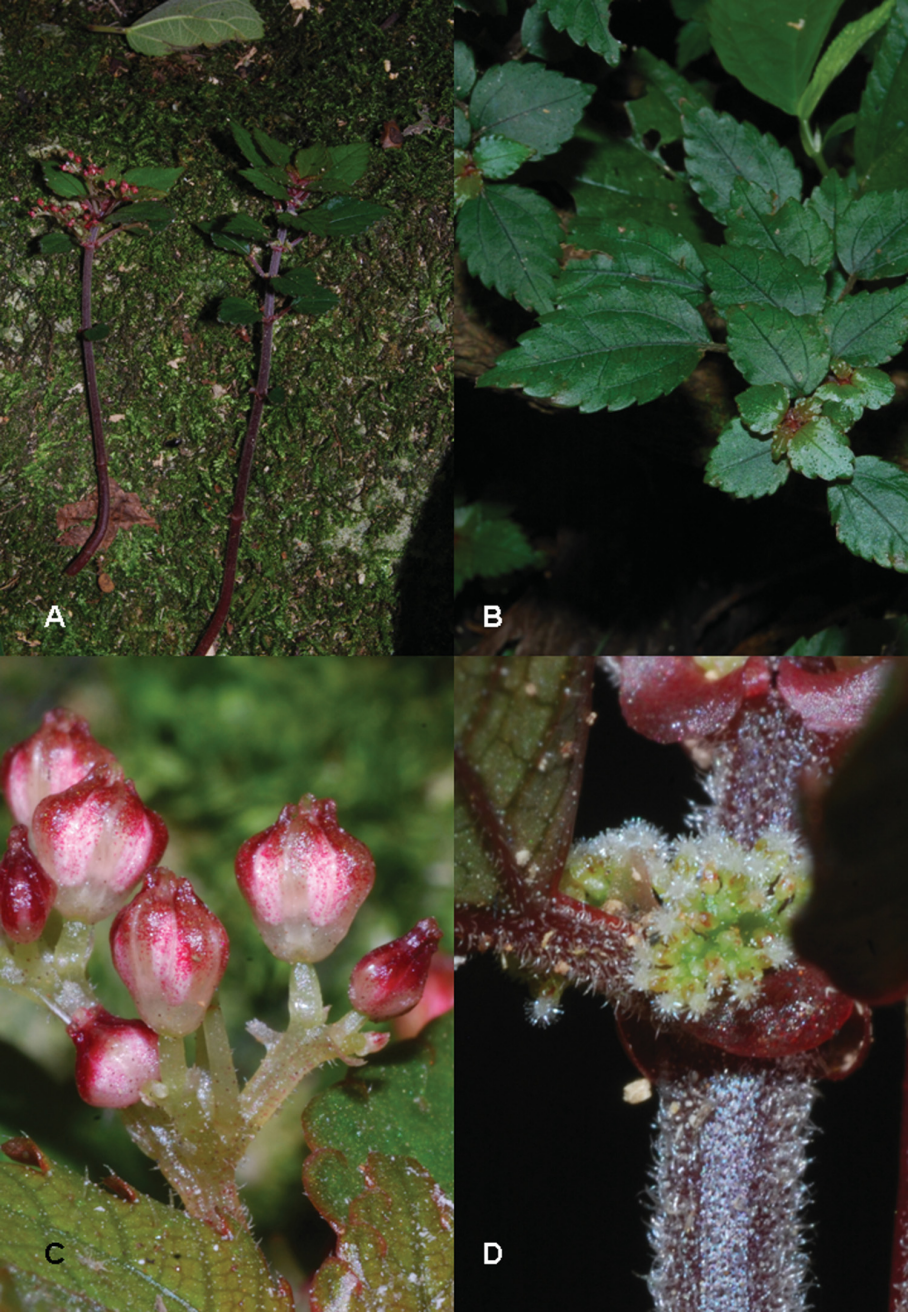
*Pilea shizongensis*. **A** Habit **B** Shoot tip **C** Close up of a staminate flowers **D** Close up of pistillate inflorescence and flowers. Based on *Monro & Wei 6727*.

Figs 3 C , 4 A–C , 5 A–D

#### Diagnosis.

Most similar to *Pilea aquarum* from which it can be distinguished by the shorter stem, serrate rather than dentate leaves, shorter stipules and glabrous pistillate tepals.

#### Type.

**China.** Yunnan: Shizong County, Feng Huang Gu gorge, 1200 m, 024°37'54.0"N, 104°14'43.9"E (DMS), 14 May 2010, *A. K. Monro & Y. G. Wei 6727* (holotype: IBK; isotypes: BM001001216, MO, PE).

#### Description.

Herb to 20 cm, epipetric and terrestrial. Stems procumbent and erect, drying dark brown, maroon to dark green when fresh, pubescent, more densely so towards the shoot tips, the hairs 0.5 mm, erect or weakly appressed, curved or crooked, orange-brown peltate glandular, cystoliths absent, the internodes 4–38 × 1.5–2.0 mm, angulate in cross-section, striate. Stipules 2.5–3.0 mm, auriculate-cordiform, drying brown. Leaves petiolate, distichous; petioles at each node unequal by ratio 1:3–4.4, 3–20 mm, pubescent, the hairs 0.25-0.375 mm, erect, strongly curved to curved; laminae at each node equal or subequal, 11–35 × 7–17 mm, ovate to broad ovate, chartaceous; 3-nerved, the lateral nerves visible for less than 2/3 of the lamina length, secondary nerves 4–6 pairs, borne 45–60° to the midrib, straight or weakly curved; upper surface drying brown or dark brown, dark green with maroon nerves and green flushed maroon when fresh, sparsely pubescent, the hairs 0.50–0.675 mm, appressed, straight or weakly curved, cystoliths absent, midrib raised; lower surface drying grey-brown when fresh, nerves densely pubescent, the hairs 0.375 mm, weakly appressed, curved, orange-brown peltate glandular, midrib and lateral nerves raised, cystoliths fusiform, randomly scattered; base symmetrical, cuneate or obtuse; margin serrate, the basal 1/4 entire; apex symmetrical, subcuspidate or cuneate. Inflorescences 4–10 per stem, unisexual, staminate and pistillate inflorescences synchronous, born on separate stems; bracts 0.375 mm; bracteoles 0.3–0.5 mm. Staminate inflorescences solitary, 17.5–25 mm, bearing 7–16 flowers in a loose cyme; peduncle 1/4 or less inflorescence length, 0.5 mm in diameter, pubescent, cystoliths absent; pedicels 0.8–1.0 mm, glabrous. Staminate flowers 1.5–2.0 × 1.5–1.8 mm immediately prior to anthesis, deep pink; tepals 4, imbricate, 1.75 mm, fused for their basal 1/4, ovate or elliptic, glabrous, the subapical appendage 0.375 mm, corniculate, glabrous; stamens 4. Pistillate inflorescences solitary, 2.0–2.5 mm, bearing 17–30 flowers in a compact cyme; peduncle 1/2 to 2/3 inflorescence length, 0.375 mm in diameter, glabrous, cystoliths absent; pedicels 0.25–0.375 mm, sparsely pubescent. Pistillate flowers 0.375–0.50 mm, tepals 3, unequal, glabrous, adaxial tepal 0.5 mm, oblong, the dorsal tepal appendage 0.375 mm, oblong, markedly thickened; the lateral tepals 0.25–0.375 mm, asymmetrically ovate. Infructescences not seen.

#### Distribution.

Yunnan Province, Feng Huang Gu gorge, ca 1200 m, in limestone karst, growing on the floor of the gorge in deep shade.

#### Etymology.

The species name refers to county of the locality of the only known collection of this species, Shizong.

#### Discussion.

Comparison of the holotype material with type specimens from the herbaria listed in the methods section recovered *Pilea aquarum* Dunn as most similar to *Pilea shizongensis*. It can be distinguished from *Pilea shizongensis* based on pubescence, leaf margin morphology and pistillate tepal and flower morphology as summarised in Table 3.

*Pilea shizongensis* falls within Weddell’s (1869) Dentatae-Gerontogeae subdivision and Chen’s (1982) Urticella Section of the genus.

There is some confusion over the delimitation of *Pilea aquarum* and this is relevant to the delimitation of *Pilea shizongensis*. It would appear that the relatively rare character trait of pubescent pistillate tepals has been overlooked by several authors and that *Pilea aquarum* *sensu strictu* encompass a relatively narrow range of morphological variation which would exclude the subspecies *Pilea aquarum* subsp. *brevicornuta* and *Pilea aquarum* subsp. *acutidentata*.

**Table 3.** XX

**Character**	*Pilea shizongensis*	*Pilea aquarum* subsp. *aquarum*
Stem height	10–20 cm	30–40 cm
Leaf margin	serrate	dentate
Pistillate tepal pubescence	glabrous	pubescent

#### Conservation status.

Using IUCN criteria (IUCN 2001) *Pilea shizongensis* is considered Endangered (E). *Pilea shizongensis* is known from a single locality (IUCN criteria D2, number of locations <5). At these localities the populations of this species comprises ca 100-200 mature individuals (IUCN criteria D1, number of mature individuals <250). Using the IUCN methodology our Global Conservation Assessment for *Pilea shizongensis* is Endangered (E) based on criteria D1 and D2: population size and number of locations combined with a plausible future threat that could drive this taxon to Endangered in a very short time. Plausible threats include the location of the only known population within a tourist site and close to the only path used by visitors to access the gorge bottom. Any expansion of the path, fire or dumping of refuse by visitors could destroy this population.

### Pilea guizhouensis

A.K. Monro, C.J. Chen & Y.G. Wei sp. nov.

urn:lsid:ipni.org:names:77123890-1

http://species-id.net/wiki/Pilea_guizhouensis

**Figure 6.**
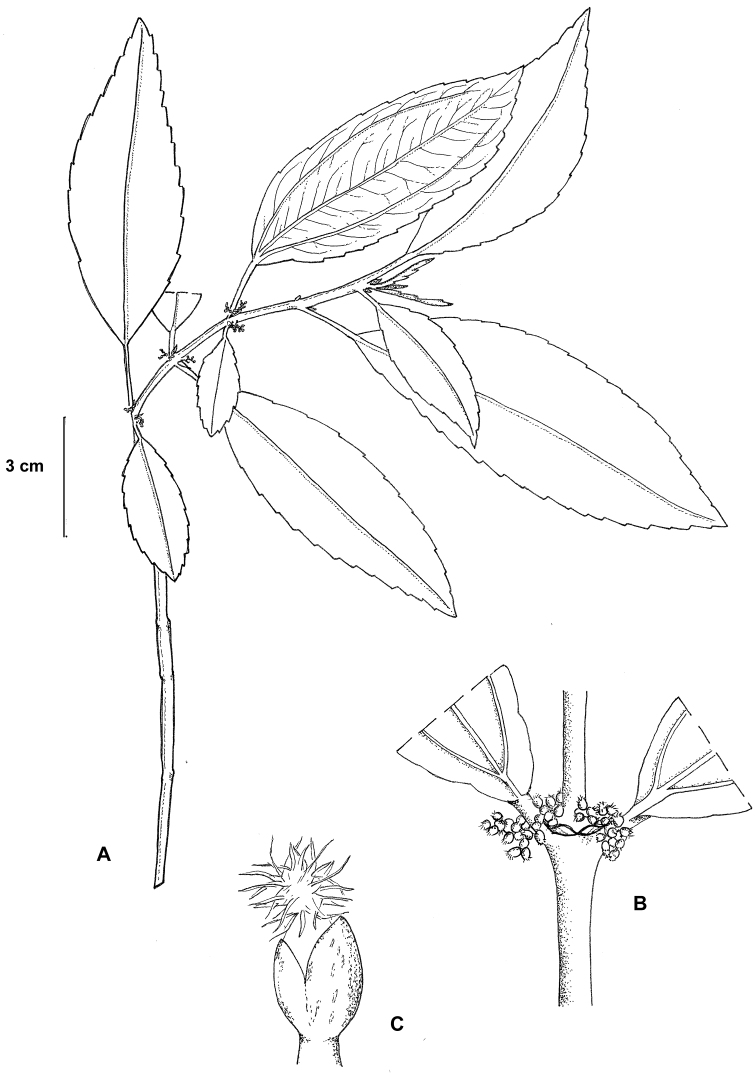
*Pilea guizhouensis*. **A** Habitat **B** close-up of pistillate inflorescence **C** close-up of a pistillate flower. Based on *Monro & Wei 6715*.

**Figure 7.**
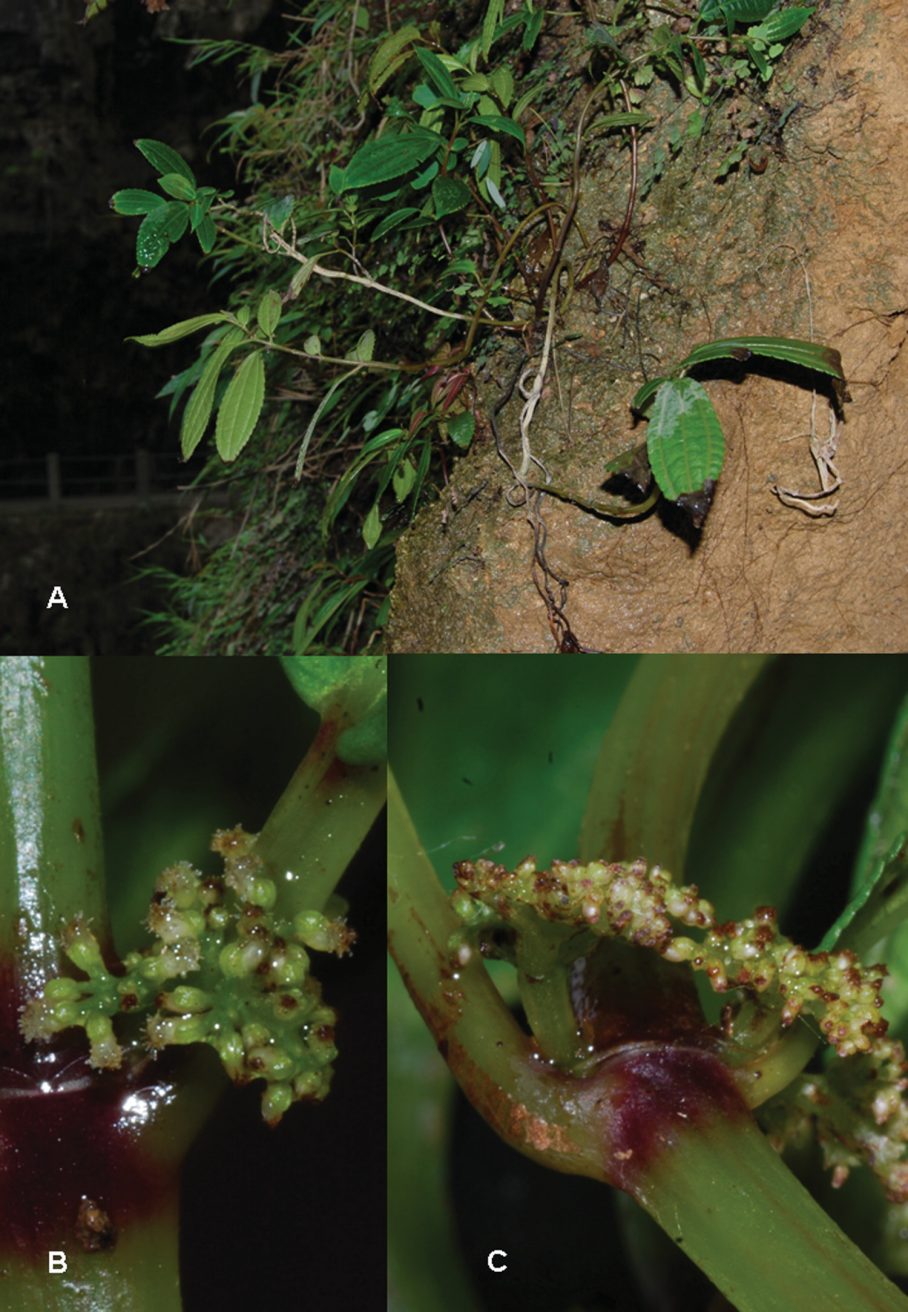
*Pilea guizhouensis*. **A** Habitat **B** close-up of pistillate inflorescence **C** close-up of a pistillate infructescence. Based on *Monro & Wei 6715*.

Figs 3 D, E , 6 A–C , 7 A–C

#### Diagnosis.

Most similar to *Pilea boniana* from which it can be distinguished by the shorter inflorescence length, staminate flowers composed of four rather than five and valvate rather than imbricate tepals, and smaller achene size.

#### Type. China.

Guizhou: Xingyi County, Xingyi City, Malinghe Gorge, 950 m, 025°08'32.9"N, 104°57'11.7"E (DMS), 12 May 2010, *A. K. Monro & Y.G. Wei 6715* (holotype: IBK; isotypes: BM001001220, MO, PE).

#### Description.

Herb or subshrub to 50 cm, epipetric or terrestrial. Stems erect, drying brown to dark brown, green and maroon at the nodes when fresh, glabrous, brown peltate glandular on young stems and flowering nodes, cystoliths elliptic or absent, the internodes 18–200 × 1.5–2.0 mm, irregularly circular and grooved in cross-section, striate. Stipules 2.0–2.5 mm, deltate, drying red-brown. Leaves petiolate, distichous; petioles at each node unequal by ratio 1:1.8-8.5, 1.5–37 mm, glabrous; laminae at each node subequal or unequal by ratio 1: 1.9–5.4, laminae 12–130 × 5–26 mm, ovate, lanceolate, asymmetrically elliptic, oblanceolate or obovate, chartaceous; 3–nerved, the lateral nerves visible for >7/8 or more of the lamina length, secondary nerves 8-14 pairs, borne 60–75° to the midrib, weakly curved; upper surface drying dark brown, dark green when fresh, glabrous, cystoliths 0.250–0.375 mm, ‘V’ shaped, midrib raised; lower surface drying brown to dark brown, pale green when fresh, glabrous, eglandular; base symmetrical or asymmetrical, cuneate or obtuse; margin discretely serrulate or serrate, the basal 1/8–1/4 entire; apex symmetrical, attenuate to acuminate. Inflorescences ca 3 per stem, unisexual, staminate and pistillate inflorescences synchronous, born on separate stems; bracts 0.75–1.0 mm; bracteoles 0.50–0.675 mm. Staminate inflorescences 15–20 mm, bearing ca 60 flowers in a loose cyme; peduncle ≤1/4 inflorescence length, 0.5 mm in diameter, glabrous; pedicels 1.0–1.5 mm, glabrous. Staminate flowers 1.5 × 1.5 mm immediately prior to anthesis, pale green; tepals 4, 1.75 mm, valvate, broad elliptic or obovate, fused for their basal 1/2, glabrous, the subapical appendage ≤0.25 mm, ridge-like, glabrous; stamens 4. Pistillate inflorescences solitary, 2.0–3.5 mm, bearing 19-42 flowers in a compact cyme; peduncle 1/4 to 1/3 inflorescence length, 0.375 mm in diameter, glabrous, cystoliths absent; pedicels 0.375–0.50 mm, glabrous. Pistillate flowers 0.50–0.75 mm, tepals 3, unequal, adaxial tepal 0.5 mm, oblong to keel-shaped, the tepal appendage 0.375–0.50 mm, appearing inflated; the lateral tepals 0.375 mm, asymmetrically ovate; staminodes not visible. Infructescences 4.0–4.5 mm; achenes 0.675 × 0.375 mm, compressed, asymmetrically ovoid, the margin narrowly thickened.

#### Paratypes.

CHINA: Guizhou: Libo County, Yaolu Town, Huangcaoping, 650 m, 025°28'25"N, 108°04'15"E (DMS, both altitude and coordinates taken from Google Earth based on label data), April 24 2005, *S. Qing & D. Londong 34* (BM001001221, PE).

#### Distribution.

Guizhou Province, Xingi County and Libo County, 650–950 m, in limestone Karst. The two localities are separated by ca 310km and represent semi-natural or naturally disturbed sites within an agricultural landscape.

#### Etymology.

The species name refers to Province from which both collections of this species are known.

#### Discussion.

Comparison of the holotype and paratype material with type specimens from the herbaria listed in the methods section recovered *Pilea boniana* Gagnep. and *Pilea rubriflora* C. Wright as most similar to *Pilea guizhouensis*. *Pilea guizhouensis* can be distinguished from *Pilea boniana* based on stipule, staminate, pistillate inflores and achene length and staminate tepal number as summarised in Table 4. *Pilea guizhouensis* can be distinguished from *Pilea rubriflora* based on internode, stipule and, staminate and pistillate flower morphology as summarised in Table 5.

Pilea *guizhouensis* falls within Weddell’s (1869) Heterophyllae-Gerontogeae subdivision and Chen’s (1982) Urticella Section of the genus.

**Table 4.** XX

**Character**	*Pilea guizhouensis*	*Pilea boniana*
Stipules	2.0–2.5 mm	ca 1.0 mm
Staminate inflorescence	15–20 mm	60–160 mm
Pistillate inflorescence	2.0–3.5 mm	60–160 mm
Staminate flower tepals	4, valvate	5, imbricate
Achene	0.675 mm	ca 2.0 mm

**Table 5.** XX

**Character**	*Pilea guizhouensis*	*Pilea rubriflora*
Internodes	1.5–2.0 mm diameter	2–3 mm in dianeter
Stipules	2.0–2.5 mm, not ribbed	ca 7 mm, longitudinally ribbed
Staminate pedicels	1.0–1.5 mm	2–3 mm
Pistillate flower tepals	3	4

#### Conservation status.

Using IUCN criteria (IUCN 2001) *Pilea guizhouensis* is considered Vulnerable (VU).*Pilea guizhouensis* is known from two localities (IUCN criteria D2, number of locations <5). These two localities are ca 320 km apart and assuming that this species occurs within the 650-950 elevation range then the Extent of Occurrence between these localities is calculated to be 12,800 km2 (IUCN criteria B1, <20,000km2). Using the IUCN methodology our Global Conservation Assessment for *Pilea guizhouensis* is Vulnerable **(VU)** based on criteria B1 with a plausible future threat that could drive this taxon to Near Threatened in a very short time. Plausible threats include the presence of a tourist trail running through the Malinghe Gorge which may be expanded, re-routed or rebuilt resulting in damage to the populations. In addition the second locality is located close to an agricultural area and is therefore vulnerable to conversion from forest to farmland.

## Supplementary Material

XML Treatment for Pilea cavernicola

XML Treatment for Pilea shizongensis

XML Treatment for Pilea guizhouensis
